# Comparative analysis of vertical double plate versus clavicle hook plate for unstable distal clavicle fractures

**DOI:** 10.1038/s41598-025-28875-w

**Published:** 2025-11-24

**Authors:** Songlin Li, Jinkang Zhang, Han Wang, Junjie Du, Bin Wang, Yufei chen

**Affiliations:** Department of Orthopaedics Air Force Medical Center PLA , Air Force Medical University , 100142 Beijing, China

**Keywords:** Distal clavicular fracture, Clavicle hook plate, Distal clavicle locking plate, Dorsal distal radius plate, Retrospective study, Health care, Medical research

## Abstract

To compare the clinical efficacy of vertical double-plate fixation (combining a distal clavicle locking plate and a radial lateral plate) versus clavicular hook plate fixation for unstable distal clavicle fractures. A retrospective analysis was conducted on 37 patients with unstable distal clavicle fractures (Neer II/V) treated at our institution from May 2015 to May 2023. Twenty-one patients underwent open reduction and internal fixation with clavicular hook plates (16 Neer II, 5 Neer V), while 16 patients received vertical double-plate fixation (12 Neer II, 4 Neer V). Postoperative evaluations included: 1) radiographic assessment of fracture healing at 1, 2, 3, and 6 months; 2) Visual Analogue Scale (VAS) pain scores during passive shoulder mobilization at 1, 2, and 4 weeks; and 3) Constant-Murley shoulder function scores and complication rates (incision infection, nonunion, acromial osteolysis, impingement syndrome) at 3, 6 and 12 months. The clavicular hook plate group (mean age 47.96±17.01 years) and vertical double-plate group (mean age 49.47±15.33 years) showed comparable demographics. All patients achieved fracture union within 3–6 months, with no implant displacement. The vertical double-plate group demonstrated significantly lower VAS scores during early rehabilitation (4.6±1.09, 4.05±0.88, 2.8±1.0 vs. 7.25±1.16, 5.9±1.12, 4.75±0.71; P<0.05). At 3, 6 and 12 months, Constant-Murley scores were markedly higher in the vertical double-plate group (84.41±4.48, 92.25±2.47, 94.55 vs. 75.35±5.92, 83.4±3.87, 88.10±2.10; P<0.05). The clavicular hook plate group exhibited higher complication rates: 4 cases of impingement syndrome, 3 of acromial osteolysis, and 5 with limited shoulder mobility—all improving after implant removal. Both techniques effectively achieve fracture union for unstable distal clavicle fractures. However, vertical double-plate fixation offers superior pain control, facilitates earlier functional rehabilitation, improves shoulder functional recovery, and reduces postoperative complications, demonstrating enhanced clinical efficacy compared to hook plate fixation.

## Introduction

Distal clavicle fractures, though accounting for only 15–28% of all clavicular fractures, are associated with alarmingly high nonunion rates of 30–45%^1,2^. This clinical challenge stems from the biomechanical instability inherent to Neer type II and V fractures, which involve partial or complete disruption of the coracoclavicular (CC) ligaments. In these injuries, the proximal fragment undergoes superior displacement due to traction from the sternocleidomastoid and trapezius muscles, while the distal fragment is pulled inferiorly by limb gravity, resulting in marked vertical instability and displacement^3,4^. Consequently, conservative management frequently leads to delayed union or nonunion, making surgical intervention the recommended approach for unstable distal clavicle fractures^3^.

Current surgical strategies include clavicular hook plates (CHP), distal clavicle locking plates (DCLP), vertical double-plate constructs, Kirschner wire tension band fixation, coracoclavicular screws, and suture-button devices for CC ligament reconstruction^5–8^. While these methods aim to restore stability, no consensus exists on an optimal technique due to trade-offs between fixation strength, complication risks, and functional outcomes. CHP and DCLP remain widely adopted for their ability to maintain anatomical reduction and fracture stability through open reduction and internal fixation (ORIF)^9^. However, the unique anatomical characteristics of the distal clavicle—its short, flat morphology and predominantly metaphyseal bone structure—pose significant challenges in achieving rigid fixation, particularly in comminuted or osteoporotic cases^10^.

To address these limitations, a novel vertical double-plate fixation strategy combining a distal clavicle locking plate and a radial lateral plate was designed. This innovative approach may offer two key advantages over conventional techniques: First, the radial lateral plate’s anatomical curvature provides improved anterior cortical contact with the clavicle, enhancing biomechanical stability. Second, the orthogonal placement of plates (anterior and superior) allows multiplanar screw fixation in the small distal fragment while resisting vertical shear forces—a critical and unique advantage unattainable with single-plate systems. This configuration theoretically enables earlier postoperative rehabilitation by minimizing micromotion at the fracture site. However, the application value of the approach has not been investigated detailedly. This study aimed to evaluate the unique clinical efficacy of this approach in reducing postoperative pain, accelerating functional recovery, and minimizing complications, thereby providing evidence-based insights for surgical decision-making.

## Materials and methods

### Study population

This retrospective study analyzed clinical data from 37 patients with unstable distal clavicle fractures (Neer type II/V) treated at the Air Force Medical Center between May 2015 and May 2023. Patients were divided into two groups based on surgical technique: 21 cases underwent clavicular hook plate (CHP) fixation (16 Neer II, 5 Neer V), and 16 cases received vertical double-plate fixation (VDPF) combining a distal clavicle locking plate and a radial lateral plate (12 Neer II, 4 Neer V). Coracoclavicular ligament reinforcement was not performed in any of the cases. All fractures were confirmed preoperatively via physical examination, radiography, or CT imaging. All procedures were performed by the same senior orthopedic surgeon. The surgical procedure involved fixation with a 2.7/3.5 mm distal clavicle locking plate, a 3.5 mm clavicular hook plate (CHP), and a 2.4 mm radial plate (the implants were all titanium alloy; Shanghai Shanyou Medical Instrument Co. Ltd., Shanghai, China).

### Inclusion and exclusion criteria

Inclusion criteria:

① Age > 18 years with acute closed distal clavicle fractures caused by shoulder trauma.
② Radiographic or CT confirmation of Neer II/V fractures.
③ Normal pre-injury shoulder function.
④ Compliance with follow-up and rehabilitation protocols.

Exclusion criteria:

① Concomitant fractures (acromion, scapula, ribs) or acromioclavicular joint dislocation.
② Prior shoulder pathologies or surgeries impairing function.
③ Open, delayed, or pathological fractures.
④ Severe systemic comorbidities contraindicating surgery.

### Ethical considerations

This retrospective study adhered to the ethical principles outlined in the Declaration of Helsinki. Written informed consent was obtained from all participants or their legally authorized representatives for participation and publication of potentially identifiable information/images in this open-access article, and the study protocol was approved by the Institutional Ethics Committee of Air Force Medical Center (approval NO. 2023-11-PJ01).

### Surgical techniques

#### Positioning and exposure

**Fig. 1:**
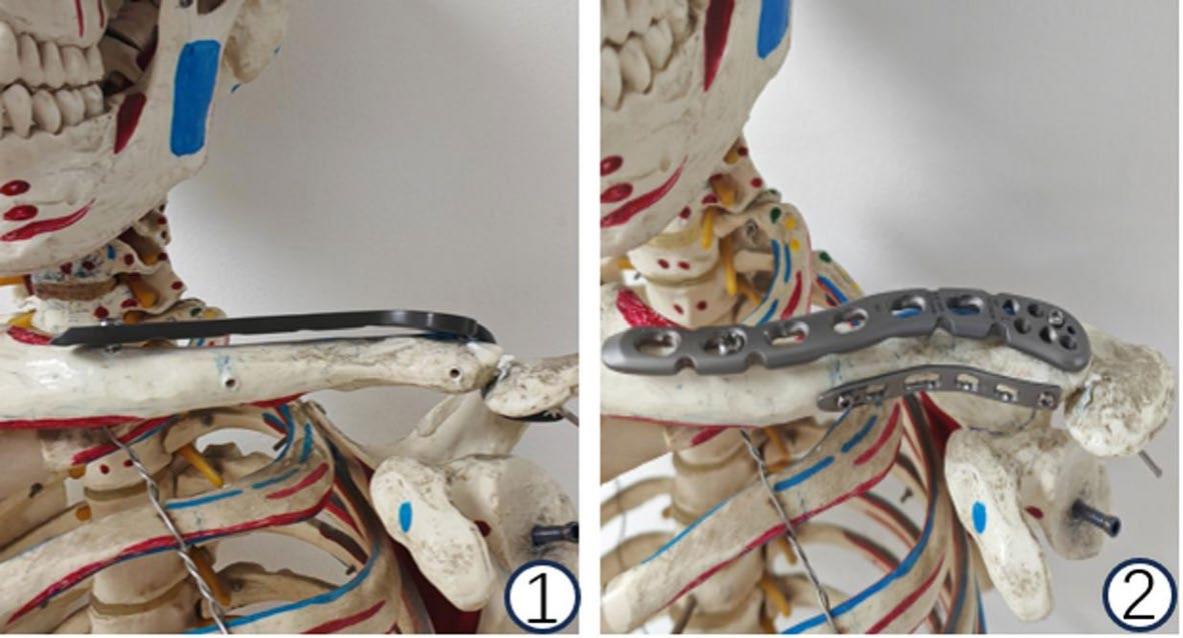
Comparative demonstration of two internal fixation devices in a model.

Patients were placed supine under general anesthesia. The affected shoulder was elevated with the head tilted contralaterally. A 5–8 cm curvilinear incision along the clavicle to the acromion exposed the fracture site, preserving the acromioclavicular joint and ligaments. Fracture hematomas and soft tissue debris were cleared (Fig. 1).

#### CHP group

A hook plate of appropriate length was inserted beneath the acromion posterior to the acromioclavicular ligament. The plate body was contoured to the superior clavicular surface. Three 3.5-mm bicortical screws were placed in the proximal fragment, with distal screw quantity determined by fragment size.

#### VDPF group

Fractures were reduced under direct vision and temporarily stabilized with Kirschner wires. A vertical interfragmentary position screw was inserted perpendicular to the fracture line. A distal clavicle locking plate was positioned along the superior clavicular margin using Kirschner wires as guides, maximizing distal fixation with 2.7-mm locking screws (≥ 3 screws) and proximal fixation with three 3.5-mm locking screws. A radial lateral plate was then placed anteriorly, secured with one 2.7-mm locking screw distally and two 2.7-mm screws proximally. Through the suture holes on the plate, the acromioclavicular ligament and surrounding soft tissues can be sutured, thereby enhancing fixation stability.

#### Intraoperative verification

Fluoroscopy confirmed anatomical reduction and implant positioning. Passive shoulder mobilization ensured fracture stability and hardware integrity. The wound was irrigated and closed in layers.

### Postoperative management

Standard wound care, analgesics, and anti-inflammatory protocols were administered. Shoulders were immobilized in a sling for 4–6 weeks. Both patient groups commenced a standardized rehabilitation program on postoperative day 2. The protocol emphasized achieving maximal functional range of motion (ROM) in all planes of shoulder movement within the patient’s pain tolerance. Exercise modalities included both active-assisted and passive ROM exercises. As pain permitted, patients progressively transitioned to active exercises. The prescribed exercise frequency was twice daily. Each session consisted of 3 to 5 repetitions of maximal attainable shoulder ROM exercises in all directions. Between postoperative weeks 6 and 8, patients advanced to include resisted strengthening exercises targeting all planes of shoulder motion. By the third postoperative month, patients were cleared to resume normal activities of daily living. CHP implants were removed upon union if symptomatic.

### Outcome measures

- Radiographic union: Assessed via anteroposterior clavicle and chest plain X-rays in the standing position at 1, 2, 3, and 6 months.
- Pain: Visual Analog Scale (VAS) scores recorded to estimate the perception of pain during passive mobilization at 1, 2, and 4 weeks. The VAS score was plotted as a 100-mm line on paper, with 2 extremes ranging from 0 (no pain) to 10 (worst pain imaginable)^11^.
- Function: Constant-Murley scores evaluating pain, mobility, strength, and daily activities at 3, 6 and 12 months^12^. The patient’s VAS and Constant-Murley scores assessments were conducted in the ward by a professional rehabilitation guidance physician with over five years of specialized clinical experience, who specializes in rehabilitation guidance within our department.
- Complications: Including infection, hardware irritation, nonunion, implant failure, acromial osteolysis, acromial impingement syndrome and shoulder stiffness. Acromial impingement syndrome defined as shoulder pain during abduction > 90° with evidence of plate-acromion contact and a positive clinical impingement sign (Neer/Hawkins). Shoulder stiffness defined as passive forward flexion < 120° or abduction < 100°, unrelated to impingement pain.

### Statistical analysis

Statistical analysis was performed using SPSS 20.0 software. Normally distributed quantitative data were expressed as mean ± standard deviation (x̄±s). Intergroup comparisons were conducted using independent samples t-test. Comparisons between two groups at different time points were analyzed by repeated measures analysis of variance (ANOVA), with post-hoc pairwise comparisons performed using LSD test. Qualitative data were compared using chi-square test. A P-value < 0.05 was considered statistically significant.

## Results

### Demographic and clinical characteristics

All patients completed follow-up (range: 10–36 months; mean: 16.93 ± 5.37 months).

- Hook Plate Group (*n* = 21): 15 males, 6 females; age range 24–80 years (mean 47.96 ± 17.01); injury mechanisms: 10 traffic accidents, 11 falls; time from injury to surgery: 4.61 ± 1.41 days.
- Double-Plate Group (*n* = 16): 11 males, 5 females; age range 23–83 years (mean 49.47 ± 15.33); injury mechanisms: 7 traffic accidents, 9 falls; time from injury to surgery: 4.38 ± 1.46 days.

No statistically significant differences were observed between groups in sex, age, injury mechanisms, time from injury to surgery or Neer fracture pattern (*P* > 0.05) (Table 1).

**Table 1:** Comparison of preoperative demographic characteristics between the two groups. (mean ± SD or n)

Group	Sex		Age, y	Cause of injury		Time from injury to surgery, d	Neer fracture pattern	
	M	F		Traffic accidents	Falls		II	V
CHP(*n* = 21)	15	6	47.96 ± 17.01	10	11 9	4.61 ± 1.41	16	5
VDPF(*n* = 16)	11	5	49.47 ± 15.33	7		4.38 ± 1.46	12	4
*P* value	0.859		0.773	0.704		0.628	0.933	

*CHP* clavicular hook plates, *VDPF* vertical double-plate fixation.

### Radiographic outcomes (Fig. 2)

**Fig. 2:**
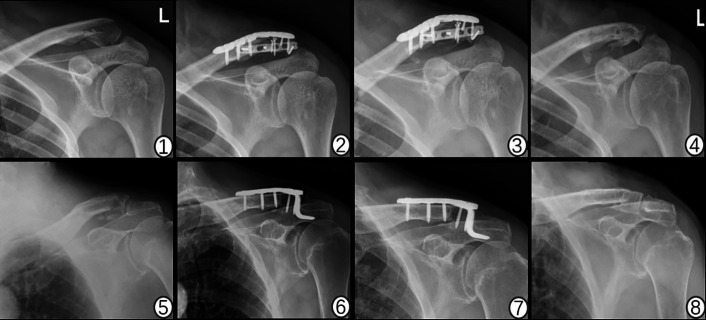
Radiographic evaluation of distal clavicle fractures treated with clavicular hook plate versus vertical double-plate. ①⑤Preoperative radiographs of Neer type V distal clavicle fractures. ①A 53-year-old male patient; ⑤A 76-year-old female patient. ②⑥Radiographs at 1 month after surgery show treatment with clavicular hook plate versus vertical double-plate.③⑦Radiographs at 3 months after surgery show good fracture healing and alignment.④⑧Postoperative radiographs at 1 year after hardware removal.

- 1-month postoperatively: Both groups exhibited satisfactory fracture alignment with stable hardware positioning.
- 2 months: Fracture lines became indistinct with visible callus formation in all cases.
- 3–6 months: Complete radiographic union was achieved in both groups, with no implant loosening or displacement.

### Postoperative pain and functional recovery

The double-plate group demonstrated superior early pain control and functional outcomes (Table 2):

**Table 2:** Comparison of VAS and Constant-Murley scores between the two groups at different postoperative follow-up time points. (mean ± SD)

	VAS				Constant-murley scores		
Follow-up	Week 1	Week 2	Week 4		Month 3	Month 6	Month 12
CHP	7.25 ± 1.16	5.9 ± 1.12		4.75 ± 0.71	75.35 ± 5.92	83.4 ± 3.87	88.10 ± 2.10
VDPF	4.6 ± 1.09	4.05 ± 0.88		2.8 ± 1.0	84.41 ± 4.48	92.25 ± 2.47	94.55 ± 1.39
F value	44.76				99.95		
*P*	*P* < 0.001	*P* < 0.001		*P* < 0.001	*P* < 0.001	*P* < 0.001	*P* < 0.001

*CHP* clavicular hook plates, *VDPF* vertical double-plate fixation.

- VAS scores during passive mobilization:
  - Double-plate: 4.6 ± 1.09 (1 week), 4.05 ± 0.88 (2 weeks), 2.8 ± 1.0 (4 weeks).
  - Hook plate: 7.25 ± 1.16 (1 week), 5.9 ± 1.12(2 weeks), 4.75 ± 0.71 (4 weeks).

Intergroup differences were statistically significant at all timepoints (*P* < 0.05).

**Fig. 3:**
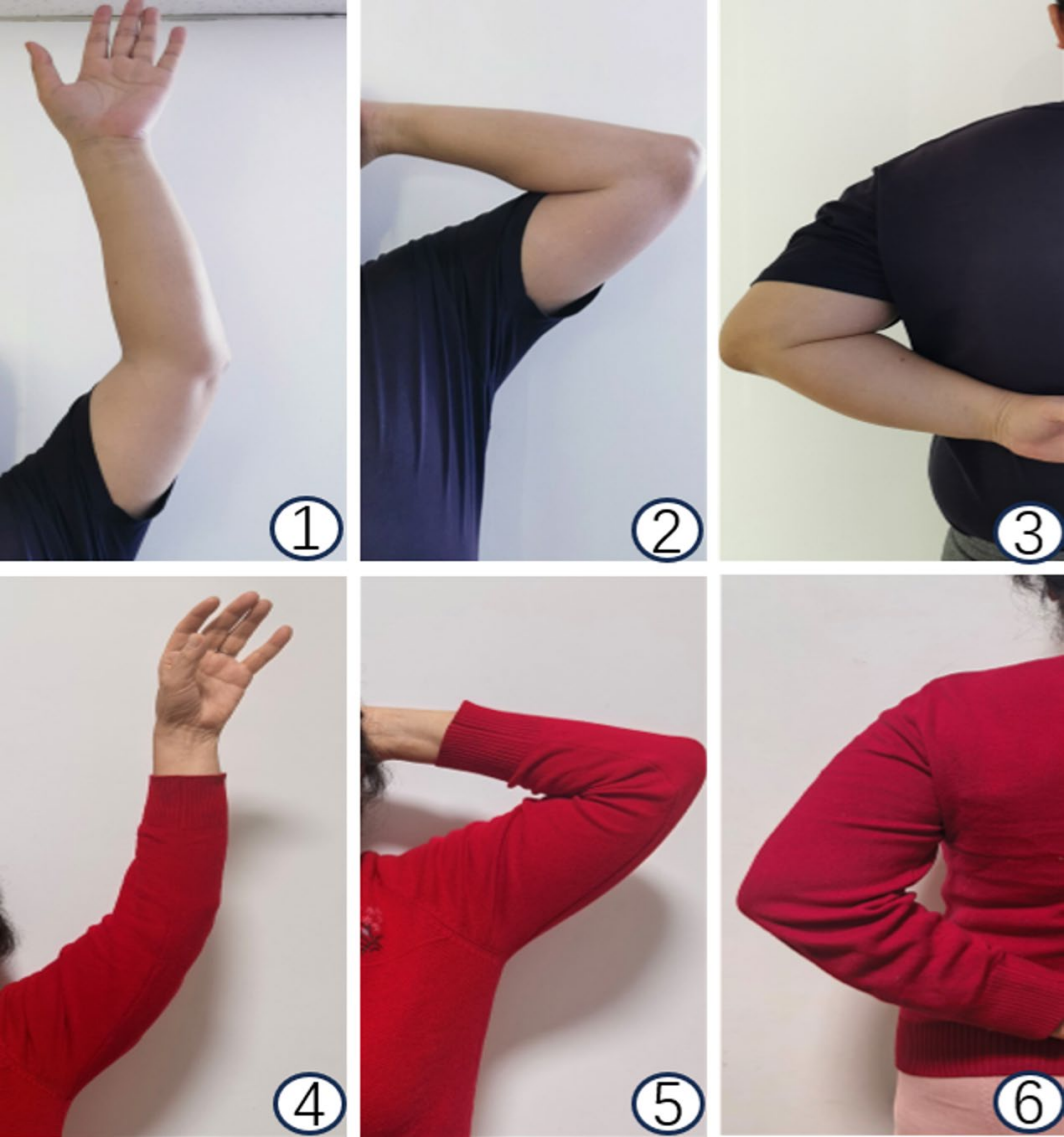
Postoperative clinical photographs showing the functional range of motion of the shoulder in two patients at the 1-year follow-up after hardware removal. ①-③: The 53-year-old male patient treated with vertical double-plate fixation. ④-⑥The 76-year-old female patient treated with clavicular hook plate fixation.

- Constant-Murley scores (Fig. 3):
  - Double-plate: 84.41 ± 4.48 (3 months), 92.25 ± 2.47 (6 months), 94.55 ± 1.39 (12 months).
  - Hook plate: 75.35 ± 5.92 (3 months), 83.4 ± 3.87(6 months), 88.10 ± 2.10 (12 months).

Significant between-group differences persisted (*P* < 0.05).

### Complications

- Common to both groups: No cases of malunion, nonunion, or hardware failure. All incisions healed primarily.
- Hook plate-specific complications:
  - 4 cases of acromial impingement syndrome.
  - 3 cases of subacromial osteolysis.
  - 5 cases of persistent shoulder stiffness.

Symptom resolution occurred following implant removal.

## Discussion

Distal clavicle fractures, accounting for 15–28% of all clavicle fractures^1,2^, are predominantly caused by falls. The modified Neer classification system categorizes these fractures into five types. Neer types I–III fractures, characterized by minimal or no displacement, are generally managed conservatively, as literature suggests that conservative treatment is suitable for stable fractures with minimal displacement. However, conservative management carries risks such as a higher incidence of nonunion, potential need for secondary surgical intervention, and a higher rate of patient dissatisfaction with early shoulder contour. Additionally, return to daily activities by 6 months post-injury is often delayed^13,14^. Neer type IV fractures, involving periosteal sleeve avulsion, occur exclusively in children. In contrast, Neer types II and V fractures are unstable due to partial or complete rupture of the coracoclavicular ligaments. The proximal fragment is displaced superiorly by the traction of the sternocleidomastoid and trapezius muscles, while the distal fragment is displaced inferiorly by the weight of the limb, resulting in vertical instability and significant displacement. For these unstable fractures (Neer types II and V), surgical treatment is generally recommended. Compared to conservative approaches, surgery is associated with fewer complications, provides sufficient stability to allow early functional exercise, and leads to more favorable functional recovery of the shoulder^13,14^. Conservative treatment for unstable fractures carries a high risk of delayed union or nonunion (30–40%)^3,4,^ necessitating open reduction and internal fixation (ORIF) for Neer types II and V fractures^10^.

Current surgical strategies include ORIF (utilizing clavicular hook plates [CHP], distal clavicle locking plates [DCLP], vertical dual plating, T-shaped plates, or Kirschner wire tension bands) and coracoclavicular ligament augmentation (with coracoclavicular screws, suture-button devices, or titanium cables). However, no standardized treatment protocol has been established^5–8^. Among these, CHP and DCLP are the most widely used in clinical practice^9^. The CHP technique involves inserting the hook of the plate beneath the acromion to elevate it, thereby compressing the distal clavicle fragment for reduction. Nils Beisemann et al.^15^ reported satisfactory outcomes in 53 patients with Neer type II fractures treated with CHP, but 11 cases (20.7%) developed complications, including surgical site infection, subacromial osteolysis, nonunion, acromioclavicular arthritis, and 10 required revision surgery. In a multicenter retrospective study comparing CHP and DCLP for Neer types II and V fractures (49 patients per group), Hiroshi Takahashi et al.^16^ demonstrated that the DCLP group achieved 100% bony union, whereas the CHP group had three nonunions (6.1%). Additionally, the DCLP group showed superior postoperative UCLA shoulder scores and fewer complications (plate displacement, subacromial impingement, loss of reduction, infection, screw breakage). Our findings align with these results: while CHP achieves bony union, it is associated with higher rates of postoperative pain, restricted shoulder mobility, and subacromial osteolysis. In contrast, DCLP avoids subacromial complications and allows earlier postoperative rehabilitation. However, DCLP alone may provide insufficient stability for small distal fragments due to limited screw fixation points.

Dual plating systems, commonly used for complex distal radius fractures and clavicular shaft nonunions^17,18^, have recently been adapted for distal clavicle fractures. Kaipel et al.^19^ first reported using dual 2.4-mm locking compression plates (LCPs) for Neer type II fractures, achieving stable fixation of small/comminuted fragments with excellent union rates, minimal pain, and functional recovery. Li Liang et al.^20^ compared four fixation methods and found that pre-contoured T-shaped plates and dual 2.5-mm LCPs reduced shoulder pain, complications, and hardware prominence while improving functional outcomes. Finite element analysis by Huang Daoqiang et al.^21^ confirmed that dual plating offers superior biomechanical stability and lower implant failure risk compared to CHP and DCLP. Although earlier studies employed 2.4-mm or 2.7-mm T-shaped plates, these lack multi-angular locking screws at the plate head. In contrast, DCLP allows divergent screw placement with angular stability, enhancing fixation strength. However, plate contouring may compromise biomechanical integrity. The DCLP’s proximal 3.5-mm screws also provide greater stability than the smaller screws (2.4–2.7 mm) of T-shaped plates. Furthermore, Through the suture holes on the plate, the acromioclavicular ligament and periarticular soft tissues can be securely anchored using high-strength sutures, thereby augmenting initial fixation stability.

The unique anatomy of the distal clavicle—flat, broad, and prone to comminution—poses challenges for fragment fixation. To address this, we employed a dual-plating strategy: the DCLP serves as the primary plate, while a radial lateral plate (placed anteriorly) acts as an auxiliary plate. Pre-bent 2.4-mm/2.7-mm plates were initially tested but exhibited reduced strength after contouring. The radial lateral plate’s anatomical compatibility with the anterior clavicle eliminates the need for bending and increases screw density in the distal fragment. This vertical dual-plate construct enhances stability, particularly in osteoporotic patients, by improving screw pullout resistance. In our retrospective study, vertical dual plating achieved stable fixation in comminuted fractures (including osteoporotic cases), tolerable postoperative pain, early functional rehabilitation, and superior shoulder function compared to CHP.

Key surgical techniques include:

1. Joint preservation: Avoid acromioclavicular joint dissection. Position the DCLP as laterally as possible, guided by two Kirschner wires marking the joint margin to maximize distal screw placement. The proximal plate may be slightly bent inferiorly for better contouring.
2. Screw trajectory control: When inserting screws into the radial lateral plate’s distal end, use Kirschner wires thinner than the drill bit to avoid collision with vertically oriented screws, preventing drill breakage or fixation failure.
3. Fragment stabilization: After reduction with reduction clamps, insert positional screws perpendicular to the fracture line to minimize fragment dispersion.
4. Subchondral screw placement: The oblique articular surface of the distal clavicle requires screw insertion into subchondral bone without joint penetration.(5) Soft tissue reattachment: The acromioclavicular ligament and surrounding periarticular soft tissues should be securely sutured through the plate’s suture holes using high-strength sutures, thereby enhancing fixation stability.
5. Soft tissue reattachment: The acromioclavicular ligament and surrounding periarticular soft tissues should be securely sutured through the plate’s suture holes using high-strength sutures, thereby enhancing fixation stability.

### Study limitations

This study has several limitations. First, its retrospective design introduces potential selection bias due to nonrandomized patient allocation. Second, the modest sample size (*n* = 37) and single-center recruitment limit the generalizability of findings. Multicenter studies with larger cohorts are needed to enhance external validity. Third, the mean follow-up duration (16.93 months) is insufficient to assess long-term complications (e.g., post-traumatic arthritis) or late hardware failure. Future research should incorporate extended follow-up periods, objective quantitative metrics (e.g., clavicular shortening, subacromial space measurements), and randomized controlled trials to validate these results.

## Conclusion

For unstable distal clavicle fractures (Neer types II and V), surgical intervention is recommended. Both vertical dual plating (combining DCLP and radial lateral plates) and CHP achieve satisfactory union rates. However, dual plating offers distinct advantages: it avoids acromioclavicular joint compromise, provides superior biomechanical stability with dual-plane screw distribution, reduces postoperative pain/complications, and facilitates early rehabilitation. These benefits, particularly evident in osteoporotic or comminuted fractures, support its clinical adoption.

## Data Availability

The datasets used and analyzed during the current study are available from the corresponding author on reasonable request.
